# Diffusion of punishment in collective norm violations

**DOI:** 10.1038/s41598-022-19156-x

**Published:** 2022-09-12

**Authors:** Anita Keshmirian, Babak Hemmatian, Bahador Bahrami, Ophelia Deroy, Fiery Cushman

**Affiliations:** 1 Department of Psychology, Harvard University, Cambridge, USA; 2 Graduate School for Neuroscience, Ludwig-Maximilians-University of Munich, Munich, Germany; 3 Faculty of Philosophy, Ludwig-Maximilians University, Munich, Germany; 4 Munich Center for Mathematical Philosophy, Munich, Germany; 5 Department of Cognitive, Linguistic and Psychological Sciences, Brown University, Providence, USA; 6 Faculty of Psychology, Ludwig-Maximilians-University, Munich, Germany; 7 Department for Psychology, Royal Holloway University of London, Egham, UK; 8 Centre for Adaptive Rationality, Max Planck Institute for Human Development, Berlin, Germany; 9 Munich Center for Neuroscience, Munich, Germany; 10 Institute of Philosophy, School of Advanced Study, University of London, London, UK

**Keywords:** Psychology, Human behaviour

## Abstract

People assign less punishment to individuals who inflict harm collectively, compared to those who do so alone. We show that this arises from judgments of diminished individual causal responsibility in the collective cases. In Experiment 1, participants (*N* = 1002) assigned less punishment to individuals involved in collective actions leading to intentional and accidental deaths, but not failed attempts, emphasizing that harmful outcomes, but not malicious intentions, were necessary and sufficient for the diffusion of punishment. Experiments 2.a compared the diffusion of punishment for harmful actions with ‘victimless’ purity violations (e.g., eating a dead human’s flesh as a group; *N* = 752). In victimless cases, where the question of causal responsibility for harm does not arise, diffusion of collective responsibility was greatly reduced—an outcome replicated in Experiment 2.b (*N* = 479). Together, the results are consistent with discounting in causal attribution as the underlying mechanism of reduction in proposed punishment for collective harmful actions.

## Introduction

In 44 BCE, Roman senators plotted Julius Caesar’s murder, collectively stabbing him more than 20 times at a senate meeting. Who, exactly, was to blame and to what extent? Many crimes like gang rape, collective hate crime, co-offending, and conspiracies are committed by groups. Understanding how blame and punishment are assigned in such group harms helps refine current models of moral judgment, and assess their correspondence with legal liability standards.

We dissociate two factors that might influence judgments of collective harm: intent to harm, and causal responsibility for it. Generally, people judge an actor as fully blameworthy if they intentionally cause harm^1–5^. Much research suggests that these two factors—intentionality and causal responsibility for harm—play dissociable roles in moral judgment^6–8^. They may influence the judgment of group actions in different, even contradictory, ways.

### Intentionality

How are intent-based moral judgments affected by the distinction between solo and group actors? One natural possibility is that group actors are held just as responsible, given that each member of the group volitionally decides to engage in transgressive behavior. Alternatively, they may also be held less responsible on the belief that they got socially “caught up” in something they would not otherwise have done^9^. In this case, group actors would receive less blame and hence punishment than solo actors committing equivalent acts.

### Causal responsibility

**Figure 1:**
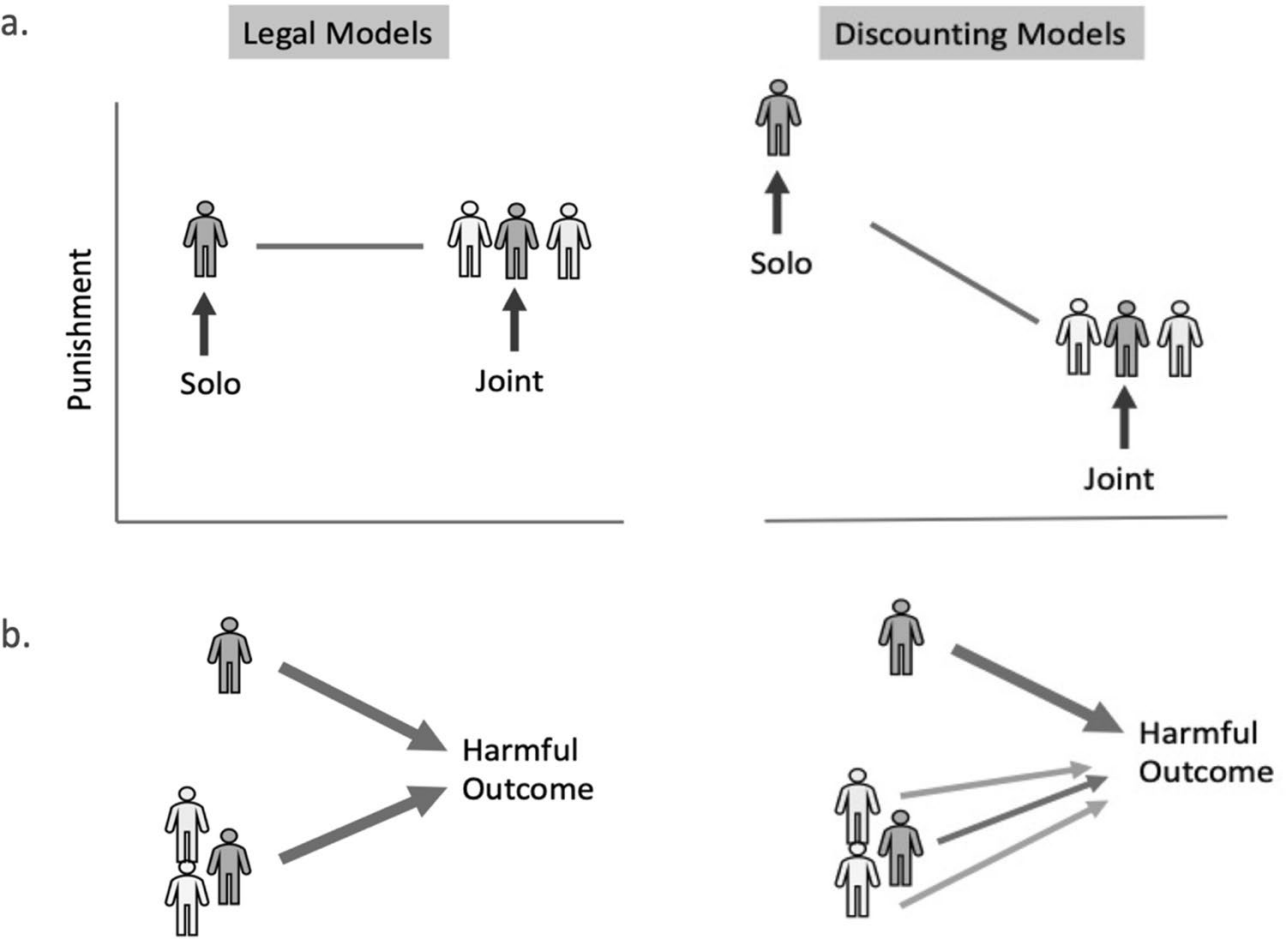
(**a**) Two models of punishment in solo and joint harmful actions: legal models suggest similar punishment for joint and solo acts. Discounting models predict less punishment in joint than solo harm violations (the diffusion of punishment hypothesis) (**b**) Causal links in two models of punishment. In legal models, all perpetrators in joint actions are causally responsible for the harmful outcome to the same degree as in solo actions. In the discounting models (the diffusion of punishment hypothesis), each individual in the group is less causally responsible for the outcome.

How will judgments of the causal responsibility of group actors compare with solo actors? Again, one possibility is that it will make no difference. Causal responsibility may be treated as categorical—one is either responsible or not^10^. Since participants in group harms are causal *contributors* to the outcome, they would be held causally responsible to the same extent as a solo actor (e.g., in felony murder^11^). For instance, in many US states (e.g., Connecticut General Statutes. tit. 53a-54c, Chapter 952; 2012), in the case of collective murder, it is argued that since the felony itself *causes* death, every participant in the felony is causally responsible for the death (Fig. 1—left panel).

Alternatively, causal responsibility may be diminished when distributed across a number of people (diffusion of punishment hypothesis; Fig. 1—right panel). This comports with several foundational ideas in the literature on causal attribution. First, “causal discounting” refers to the idea that causal attributions to one variable are diminished as other contributing variables are introduced^12,13^. Second, “overdetermination” happens when an effect would have occurred without the contribution of any sole individual. In such cases, people are likely to perceive each individual as less causally responsible^14^. Similarly, the degree to which the individual has causal control over the outcome may be diminished in collective violations, and so causal power theory would suggest diminished attributions of causal responsibility^15^. Because punishment judgments are sensitive to attributions of causal responsibility for harm^6^, these notions predict diminished punishment for group actors.

### Existing research

Evidence on the punishment of groups compared to solo actors is mixed. An archival study suggested that judges give harsher sentences to lone offenders compared to group offenders, controlling for the crime^16^. However, a follow-up experiment on hypothetical robberies failed to find corroborating evidence, a result ascribed to its small sample size^16^. More recently, researchers investigated second-party punishment in fairness-based group games but found no difference between proposed punishment for lone fairness violators compared with collective ones^17^. However, second-party punishment introduces unique self-oriented emotions and motives, such as retaliation. These may bias second parties to attribute heightened intent and causal responsibility, even for groups, which could mask the diffusion of punishment. Another study about punishing cheaters showed that group violators are considered less dishonest than individual ones, but differences in judgments of deserved punishment were not statistically significant (*p* = 0.08)^18^.

A key limitation of prior studies is that they cannot dissociate the potentially divergent roles of intent-based and responsibility-based processes in deserved punishment judgments. To disentangle these, in Experiment 1, we compared accidental harm (where there is no malign intent, but causal responsibility is preserved) and attempted harm (where the intent is preserved, but no harm is caused). In Experiment 2, we investigated cases of collective “harmless” purity violations, such as disrespecting the deceased, and compared them to collective harmful actions. Like attempted harms, these preserve the element of volitional action going against moral norms while eliminating any relevant question of causal responsibility.

## Experiment 1

Experiment 1 tested whether a third party punishes an individual less if she inflicts a harmful outcome on a victim as part of a group, rather than acting alone. We expected less punishment assigned to individuals in a harmful joint action compared to harmful solo actions, due to a diffusion of causal responsibility for the harm. However, when the group *intended* to cause harm but no harm ensued, we did not expect to see any difference between punishment in solo versus joint actions.

We employed a 2 × 2 × 2 design with three factors: Collectivity, Malicious Intent and Causation of Harm. Collectivity was a between-subjects factor, while Malicious Intent (henceforth called ‘Intent’ in short) and Causation of Harm (henceforth called ‘Causation’ in short) served as within-subject factors. By independently manipulating agents' Intent (absent vs. present) and Causation (absent vs. present), we can differentiate between the effects of Collectivity on Intent- and causal responsibility-based processes of moral judgment.

### Methods

#### Participants

One thousand and seventy-five participants were recruited via Amazon's Mechanical Turk. Thirty-seven participants were excluded for having duplicate IDs. We used a data-driven Mahalanobis Distance measure^19^ to identify non-human participants and inconsistent or inattentive responses (see Supplementary Material—Sect. 1.1). This step resulted in excluding 36 participants. We replicated the main results including those who failed the Mahalanobis exclusion criterion (see Supplementary Material—Sect. 1.1.2, Table S3). The final sample of 1002 US residents (452 males, eight choosing the "other" option) had an average age of 29.29 years (*SD* = 7.46, range: 18 to 64).

#### Material and procedure

Each participant was randomly assigned to one of two Collectivity conditions (joint or solo action) and read four moral scenarios in which a character committed an act either as part of a group (joint action) or alone (solo action). The dependent measure was always the deserved punishment for a given character on a 7-point scale (1 labeled as "not at all", 4 as "somewhat", and 7 as "a lot").

**Figure 2:**
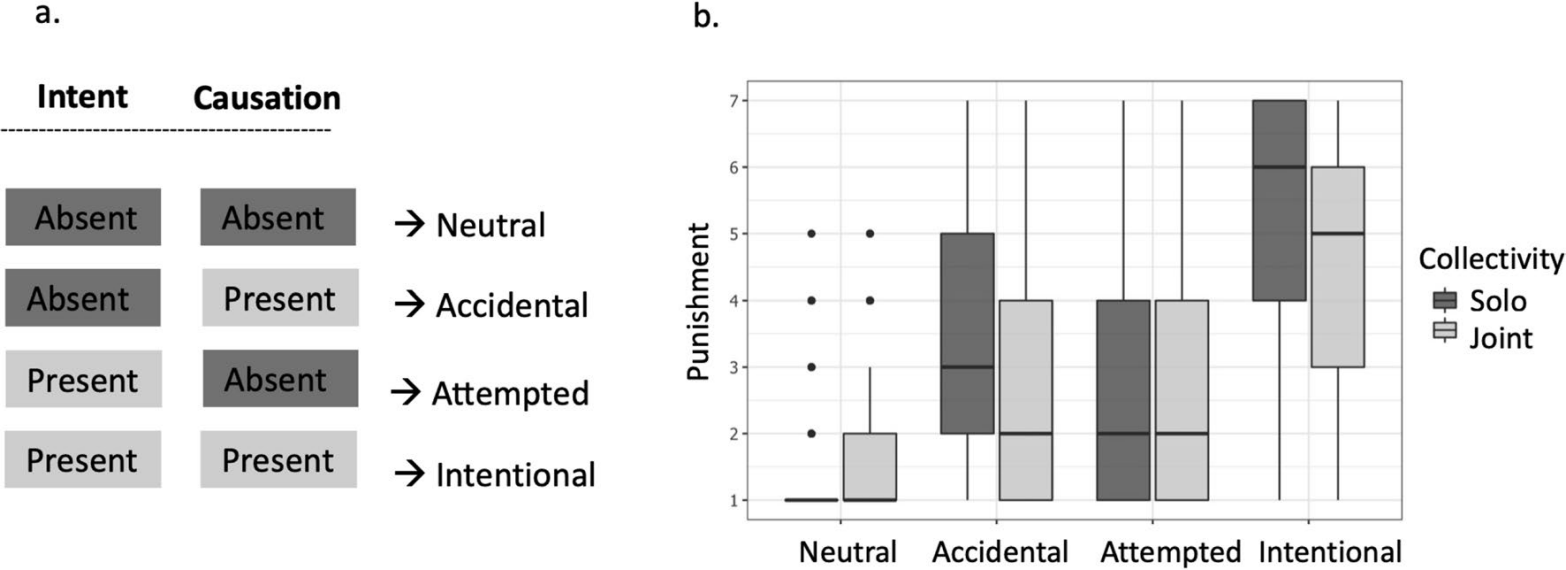
(**a**) The four experimental conditions as the outcome of a 2 × 2 design crossing Malicious Intent (absent vs. present) and Causation of Harm (absent vs. present). (**b**) Box-and-whisker plot of punishment ratings as a function of Collectivity (different colors) across neutral, accidental, attempted, and intentional actions (horizontal axis). The box represents the middle 50% of scores. The thick horizontal line within each box represents the median. Upper and lower whiskers show the range of scores in the highest and lowest quartiles. The dots represent outliers.

Intent (absent vs. present) and Causation (absent vs. present) were crossed within subjects across the four scenarios (see Fig. 2a). In neutral conditions, the agent(s) acted with no malign intention, and caused no harm. Accidental conditions involved an unintended death following the described action. In the attempted and intentional cases, the agent(s) acted with malign intent, either failing or succeeding in murdering another person. The following is an intentional, solo, harmful action scenario adapted from a previous study^8^ (see Supplementary Material—Sect. 3.1 for full scenario texts):

*Stacey* and Kate are friends and decide to go rock climbing. They are going to use new harnesses to scale a gigantic cliff.

Kate starts to put on one of the new harnesses. The clamp on the new harness is subtly flawed, so the whole harness is incredibly unsafe to use.

Because the clamp on the harness does not audibly click into place, *Stacey realizes* that the new harness is malfunctioning and may not be safe to use.

*She straps Kate into the harness and asks Kate to go first.* Partway up the cliff, the harness gives way, causing Kate to fall and die.

The three sentences in italics were substituted in joint action conditions with statements about "Stacey, Anita, James, and Kate" instead, implicating the first three characters in the harm inflicted on the last-named individual.

A random pairing of stories was first created for within-subject manipulations and then counterbalanced across participants. The order of scenarios was randomized. Demographics followed the last vignette, including age, gender, political orientation (from 1 denoted as "very liberal" to 7 marked as "very conservative"), ethnicity, and education level.

### Results

Figure 2b shows the results of Experiment 1. Statistical analysis was conducted using R (https://www.r-project.org/), employing generalized mixed-effects models appropriate for our design's hierarchical structure. Since our dependent variable has a Likert scale, we employed ordinal logistic mixed-effect models using the ‘ordinal’ package^20^.

Punishment ratings for intentional harm were significantly higher than accidental and attempted harm, and ratings in the mentioned conditions all exceeded those for the neutral condition, showing that the Intent and Causation manipulations worked as expected (see Supplementary Material—Sect. 1.2.2, Table S2).

To test the diffusion of punishment hypothesis, we modeled punishment judgments using an ordinal mixed-effects model. We included Collectivity (solo vs. group), Intent (present vs. absent), and Causation (present vs. absent) as fixed effects, along with all possible interactions. We included participant and vignette as random intercepts, along with the ‘maximal’ random slopes structure advocated in prior research^21^. We then performed a series of model comparisons, contrasting this full model with sparser models omitting fixed effects of interest. Model comparison favored the variant including all three factors (see Supplementary Material—Sect. 1.2.2).

The interactions between Collectivity and Causation (*b* = 1.412, *SE* = 0.195, *z* = 7.245,* p* < 0.001, two-tailed test), and between Collectivity and Intention (*b* = 1.005, *SE* = 0.195, *z* = 5.159, *p* < 0.001, two-tailed test) were significant. To better interpret the results, we computed contrasts over estimated marginal means using the ‘emmeans’ package in R^22^. Pairwise comparison (adjusting for multiple comparisons using the Tukey method) showed less assigned punishment for characters involved in joint actions compared to solo actions for intentional (*b* = 0.574, *SE* = 0.129, *z* = 4.451,* p* < 0.001, two-tailed test) and accidental killings (*b* = 0.436, *SE* = 0.128, *z* = 3.412, *p* < 0.015, two-tailed test), but not significant in failed murder attempts (*b* = 0.029, *SE* = 0.129, *z* = 0.229, *p* = 1, two-tailed test). Since we predicted a null effect for failed attempts, following Aczel et al.’s^23^ method, a Bayesian mixed-effect analysis was performed using the ‘brms’ package in R^24^ to confirm the pattern of results (intentional: *BF*_10_ = 498.32, *CI*_*9 5*_ = [0.29, 0.69]; accidental: *BF*_10_ = 21.74, *CI*_*95*_ = [0.12, 0.52]; attempted: *BF*_10_ = 0.05, *CI*_*95*_ = [-0.17, 0.22]; see Supplementary Material—Sect. 1.3, Table S4). In addition, to ascertain that the effect size we observe in failed attempts was small or close to zero, we performed an equivalence test using the ‘TOSTER’ package in R^25^. The equivalence test further confirmed that the distribution of punishment in joint vs solo attempted murders were equivalent (*z* = 7.770,* p* < 0.001, two-tailed test), indicating no significant difference between the two conditions. Unexpectedly, protagonists in neutral conditions received harsher proposed punishment for joint compared to solo actions (*b* = 0.976, *SE* = 0.166, *p* < 0.001, two-tailed test; see Fig. 2.a and Table S2 in Supplementary Material—Sect. 1.2.2). This effect was not predicted. Comparisons of specific items can be found in Supplementary Material—Sect. 1.4, Figure S2.

### Discussion

We found a robust reduction in proposed punishment across instances of intended and accidental harm when perpetrators acted as part of a group rather than lone agents. The contrast between these results and previous studies^16,17^ may be attributed to the more representative range of clearly and more strongly harmful Causations (i.e., death) represented in our materials. That no diffusion of punishment was observed for attempted harm suggests that diffusion of punishment depends on the discounting principle involved in *causal* attribution of harmful *outcomes* rather than intentions.

## Experiment 2

Not all acts deemed immoral involve causing harm. 'Victimless' purity violations are condemned on the basis of a moral norm violation rather than harmful causation^25^. They are judged based on perpetrators' impact on themselves rather than victims^27–29^, and elicit disgust only when people judge moral character rather than outcomes^30^. If the diffusion of punishment results from a discounting principle in causal attribution, it would only apply to actions that cause harmful outcomes. Therefore, we expected it to be weaker for judgments of purity violations.

We test this prediction in Experiment 2 by directly comparing diffusion of punishment in scenarios involving harm and purity violations. Unlike most previous studies, instead of assuming that harm scenarios induce a sense of harmfulness alone and the purity vignettes only a sense of disgust, we asked participants to rate how harmful or gross they found the protagonist(s)' action in all scenarios, examining their evaluation of the causation more directly.

### Experiment 2.a

#### Methods

##### Participants

A target sample size was predetermined using a Monte Carlo simulation following guidelines provided by DeBruine and Barr^31^ (see Supplementary Material Sect. 2.5.1). The final sample consisted of 752 US and UK residents (331 females; three others, age: *M* = 28.03 years, *SD* = 6.63, range: 18 to 60) recruited through Prolific Academic (https://www.prolific.co/) and compensated for their time. Twenty-six participants were excluded for having the same Prolific IDs as those from a pilot study. To increase precision, we used data-driven methods (as in Experiment 1) to exclude inattentive responders. Another 39 were excluded for failing attention checks—they assigned 0 to 49 (on a 100-point scale) blame to a person who "destroys the entire planet" (*n* = 18), and 51 to 100 for someone who "gives money to a charitable organization" (*n* = 21).

##### Materials and procedure

Experiment 2a compared judgments for less grave *harm* than in Experiment 1 (e.g., intentionally breaking someone's leg) with *purity* violations (e.g., masturbating over a grave). Collectivity was manipulated as before. The moral Domain (harm vs. purity) was manipulated within subjects. Hence, each subject responded to four scenarios, presented to her in two blocks (for harm and purity, respectively). The scenarios were randomly chosen from a battery of 8 items, counterbalanced across participants. The order of blocks and the items within each block were randomized and counterbalanced. Four items were adapted from a previous study on harm^8^, while four items were original scenarios representing purity violations (some inspired by a previous study^32^; see Supplementary Material—Sect. 3.2 for the full text of scenarios). The items were matched for severity of joint vs. solo action in a pilot study.

For instance, a purity violation in the solo condition would read:

Dan's favorite singer has died and has been buried in a nearby cemetery. He always had wild fantasies about the singer, and one night, he forms the following plan: He enters the cemetery late at night and goes to masturbate over the singer's grave, making sure he cannot be seen. After that, he ensures that the grave is clean and exactly as it was before and leaves.

The same scenario in the joint condition would introduce Dan, Ray, and Carl as friends who collectively committed the act.

To allow more precise judgments by providing more response options, we measured proposed punishment on a 100-point Likert scale. Zero to 50 was labeled as mild and 50 to 100 as severe punishment. Perceived Harmfulness and Grossness were measured on similar 100-point scales. Judgments of blameworthiness (hereafter “Blame”) were also gathered in Experiment 2.a to rule out an alternative explanation for the diffusion of punishment: if the practical difficulty of punishing multiple violators compared with a single actor is behind the diffusion, Blame judgments that are more removed from such practicalities would not show the effect. The results were in line with our non-pragmatic interpretation of diffusion, despite some differences with punishment ratings (see Supplementary Material—Sect. 2.3).

Like Experiment 1, we employed generalized ordinal mixed-effects models appropriate for our design’s hierarchical structure. Since our dependent variable was on a 100-point scale, we employed linear mixed-effect models through the ‘LME4’ package in R^33^**.** Other aspects of the design were identical to Experiment 1.

#### Results

We designed the scenarios with the goal of minimizing perceived harm in Purity conditions and perceived grossness in Harm conditions. However, no scenario garnered an average perceived Harmfulness rating of close to zero. Therefore, we first tested whether perceptions of harm and purity violation show the expected results, regardless of researcher-assigned labels for scenario Domains. Punishment remained the key outcome measure throughout this analysis.

We modeled Harmfulness and Grossness ratings using a linear mixed-effects model with Collectivity (solo vs. group) and Domain (purity vs. harm) as fixed effects, along with their possible interactions. Participant and vignette were included as random intercepts, along with ‘maximal’ random slopes^21^. Model comparisons contrasting this full model with sparser models ensued. The full model showed the best performance. Contrasts over estimated marginal means were calculated using the ‘emmeans’ package^22^.

**Figure 3:**
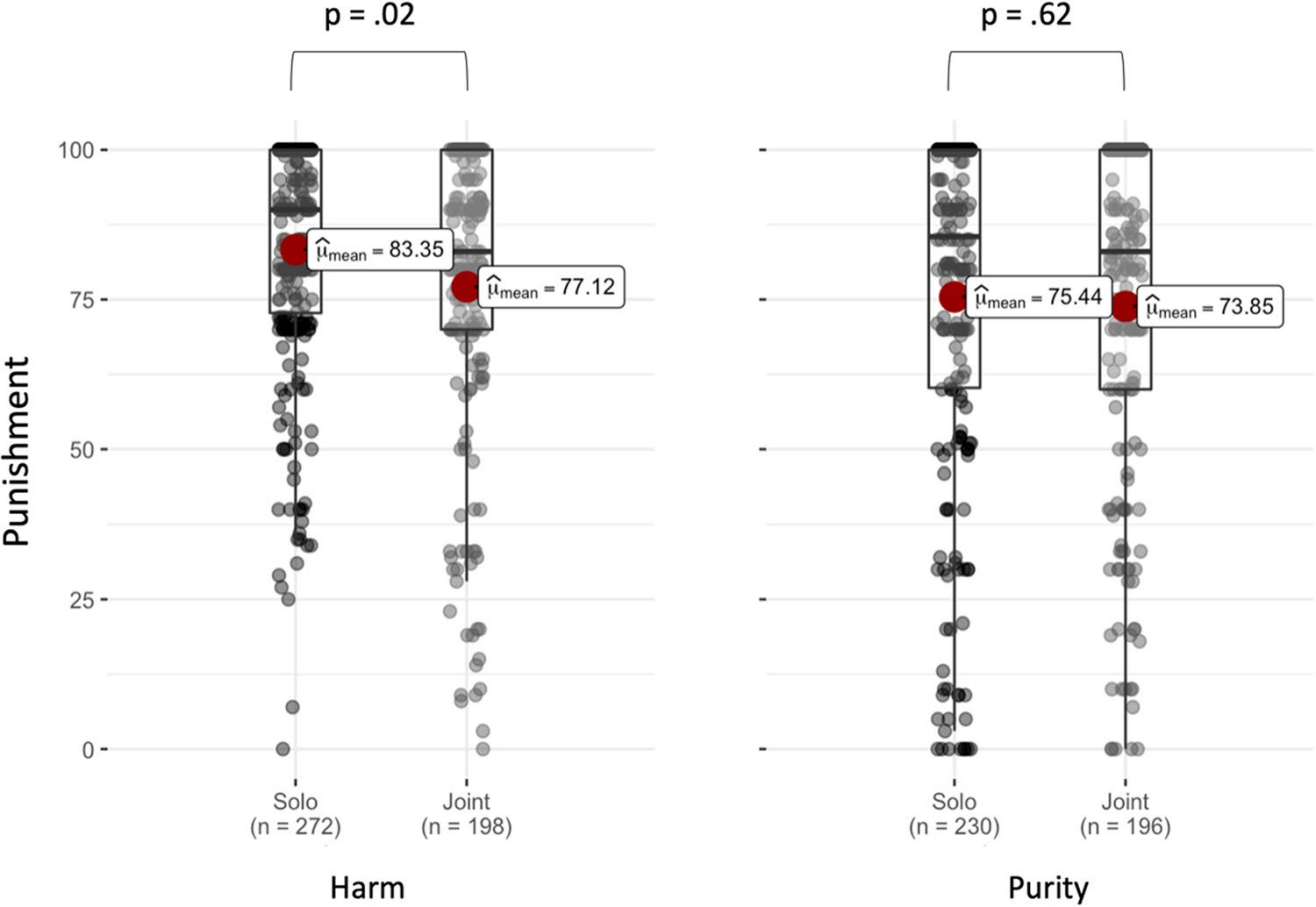
Harmfulness (left) and Grossness (right) ratings are matched across Joint and Solo actions but significantly different across Domains: Harmfulness is higher in Harm (left), and grossness is higher in Purity (right) scenarios. Graph conventions are the same as Fig. 2.

As expected, across joint and solo norm violations, significant effects of Domain were found for Harmfulness (*b* = 40.032, *SE* = 4.686, *z* = 8.542, *df* = 6.372, *p* < 0.001, two-tailed test) and Grossness (*b* = 41.454, *SE* = 2.430, *z* = 17.059, *df* = 7.366, *p* < 0.001, two-tailed test), indicating that participants perceived Harm scenarios as significantly more harmful (and less gross) than Purity scenarios (see Fig. 3). No significant effect of Collectivity was found for Harmfulness (*b* = 0.531, *SE* = 1.497, *z* = 0.354, *df* = 1324.678, *p* = 0.722; two-tailed test; *BF*_10_ = 0.754,*CI*_*95*_ = [-1.68, 1.44]) or Grossness ratings (*b* = 0.573, *SE* = 1.727, *z* = 0.087, *df* = 1150.680, *p* = 0.704; two-tailed test; *BF*_10_ = 0.76, *CI*_*95*_ = [− 1.57, 2.26]), whether using linear mixed-effects analysis^33^ or its Bayesian counterpart^24^. Because the interaction between Domain and Collectivity was not significant in Harm (*b* = 1.270, *SE* = 1.649, *z* = 0.77, *df* = 2053.909, *p* = 0.442 two-tailed test) or in Purity (*b* = 1.408, *SE* = 1.696, *z* = 0.830, *df* = 2052.974, *p* = 0.406, two-tailed test), we did not further calculate a main effect of Domain. In addition to establishing the adequacy of our item construction, these results support the Moral Foundations account of disparate moral domains for harm and purity^34^.

**Figure 4:**
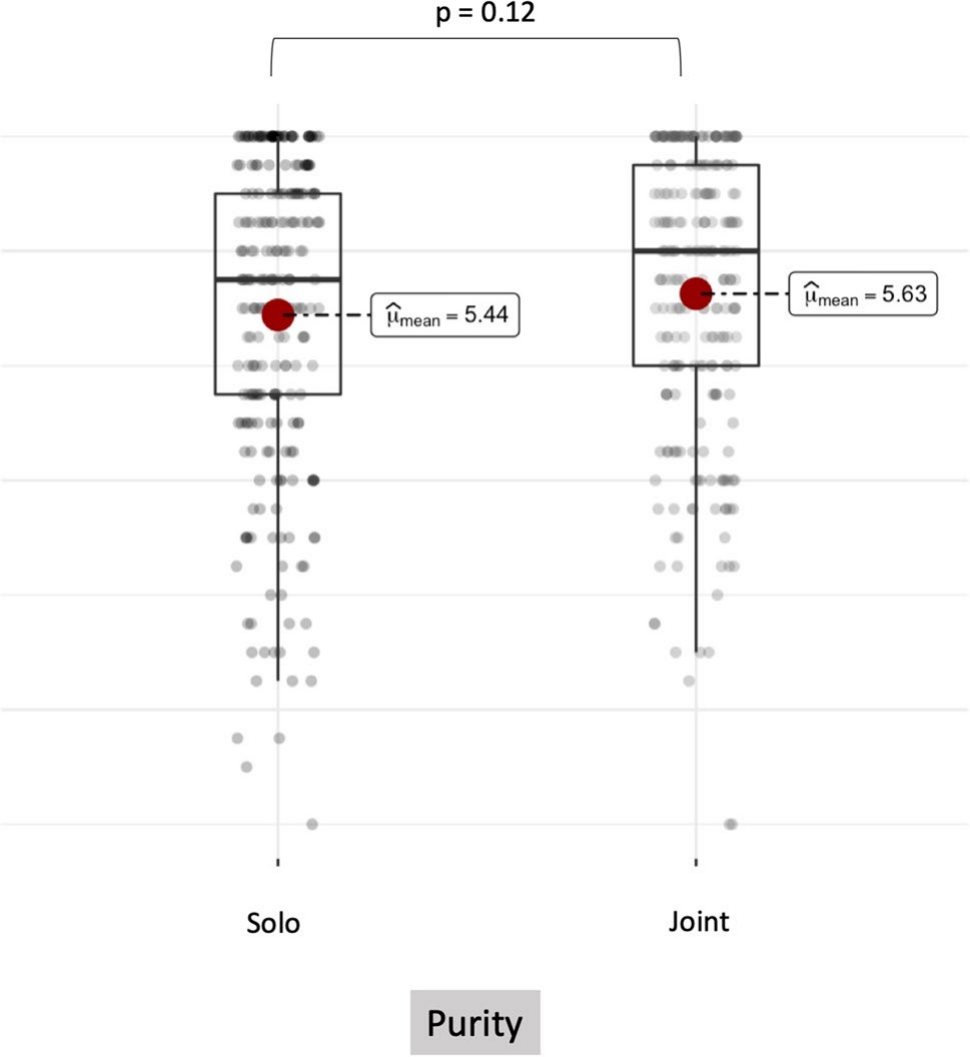
In the first blocks, participants punished individuals for Solo actions (dark grey) more than Joint actions (light grey) in Harm scenarios (left) but not in Purity conditions (right).

We then investigated the interaction between Collectivity (Solo vs. Joint) and Domain (Purity vs. Harm) in a linear mixed-effect model predicting punishment ratings, including participant and vignette as random intercepts along with ‘maximal’ random slopes^21^(see Supplementary Material—Sect. 2.1.1). There was a main effect of Collectivity (*b* = 3.364, *SE* = 1.519, *z* = 2.214, *df* = 1139.669, *p* = 0.027, two-tailed test) but the predicted interaction with domain was not significant (*b* = 0.181, *SE* = 1.480, *z* = 0.122, *df* = 2053.622, *p* = 0.902, two-tailed test). Given the within-subjects blocked design, we investigated possible spillover effects in which participant responses to the first block (of, e.g., harm cases) would affect their responses in the second block (of, e.g., purity scenarios). This might have the effect of deflating diffusion of punishment in harm cases and inflating it in purity cases. To confirm the dissociation of Domains, we dissected the first blocks into Harm and Purity datasets. An exploratory mixed effect analysis (not preregistered) with a model similar to the above –but only including responses to the first blocks—showed the predicted significant diffusion of punishment in Harm (*b* = 6.005, *SE* = 2.648, *z* = 2.266, *df* = 232.942, *p* = 0.024, two-tailed test), but not Purity blocks (*b* = 1.742, *SE* = 3.503, *z* = 0.497, *df* = 209.431 , *p* = 0.619, two-tailed test) (see Fig. 4; for more details see Supplementary Material—Sect. 2.1.1). To further confirm that the effect size we observed in Purity cases was small or close to zero, we performed an equivalence test^25^. As predicted, the equivalence test was significant (*z* = 4.58,* p* < 0.001, two-tailed test), showing that punishment distributions in Joint and Solo violations of Purity are equivalent in the first blocks.

**Figure 5:**
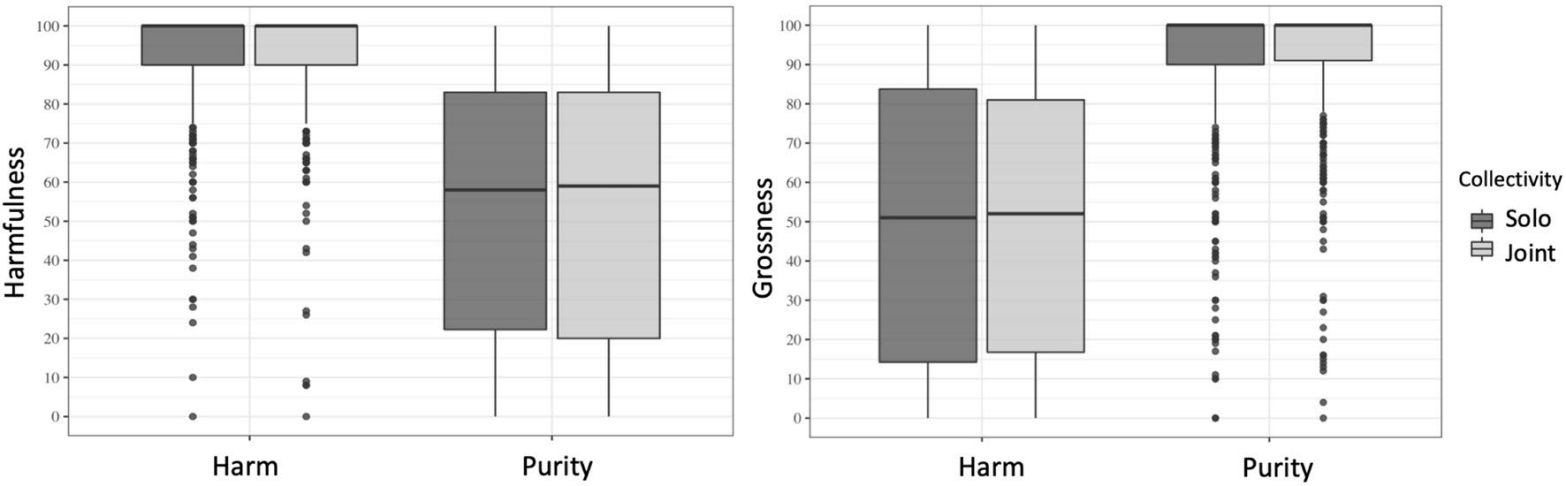
No difference in punishment judgments was observed between Solo and Joint Purity violations in Experiment 2.b.

We used this dataset to test the Collectivity by Domain interaction in a linear mixed-effect model where Domain (Harm vs Purity) was introduced as a between-subject factor. Using the combined Harm and Purity dataset, rather than dissecting the first blocks into two independent sets, allows us to maintain greater statistical power. The predicted interaction with Domain was not significant in the combined first-block dataset *(b* = 4.251*, SE* = 4.347*, z* = 0.978, *p* = 0.328*, df* = 442.213, two-tailed test*)*. A post-hoc power simulation based on the observed parameters showed that our sample size was inadequate to reliably detect a plausible effect size for the interaction (see Supplementary Material—Sect. 2.5). To ensure adequate power and confirm that carryover effects are responsible for the observed diffusion in the Purity domain, we conducted a pre-registered replication with only purity violations in a between-subjects design.

### Experiment 2.b

#### Methods Exp 2.b

##### Participants

A target sample size of 500 was predetermined using a Monte Carlo simulation via the ‘SIMR’ package in R^35^. We determined the sample size with 93.00% (91.24, 94.50) power to detect the main effect of Collectivity in the Harm domain with the same parameters obtained from the main regression model of punishment in Experiment 2.a. We recruited 526 US and UK residents to ensure that, after exclusions, the sample size will be close to the target. Participants were recruited through Prolific Academic (https://www.prolific.co/) and compensated for their time. Thirty-nine entries were excluded for having Prolific IDs that duplicated those from a pilot study. Attention checks and their results were as in Exp 2.a. Eight participants were excluded for failing attention checks. The final sample consisted of 479 (312 females; five others, age: *M* = 27.53 years, *SD* = 6.57, range: 18 to 62).

##### Materials and procedure

The number of protagonists was manipulated as in Experiment 2.a, but only one moral Domain (Purity) was provided to the subjects. Each participant responded to four fully randomized scenarios, all from the Purity domain. The scenarios were identical to Experiment 2.a. We measured deserved punishment after each scenario on a 7-point Likert scale similar to Experiment 1 since the 100-point scale had no impact on the results in Experiment 2.a.

#### Results

A linear mixed-effect analysis was performed with Collectivity (Joint vs. Solo) as a fixed factor, and participants and vignettes as random factors. Pairwise comparison indicated that judgments were similar in Joint and Solo purity violations (*b* = 0.175, *SE* = 0.119, *z* = 1.464, *df* = 476.997, *p* = 0.143; two-tailed test), which was confirmed by Bayesian mixed-effects analysis (*BF*_10_ = 0.377, CI_*95*_ = [− 0.429, 0.041]) (see Fig. 5 and Supplementary Material—Sect. 2.2). To further confirm that the effect size we observed in Purity cases was small or close to zero, we performed an equivalence test. Using TOSTER package in R^25^. As predicted, the equivalence test was significant (*z* = 8.738,* p* < 0.001, two-tailed test), showing that punishment distributions in Joint and Solo violations of purity are equivalent.

#### Discussion

In Experiment 2.a, we found a stronger diffusion of punishment in the Harm domain, along with evidence that any diffusion in Purity scenarios may be due to carryover effects. Confirming this interpretation, our preregistered Experiment 2.b found no diffusion of punishment for actions deemed impure but harmless, despite ample power. This suggests that punishment is diffused only when the collective action contains a causal link to a harmful outcome, and is absent for victimless moral violations.

## General discussion

Though group immoral actions are commonly performed, punitive reactions to them are rarely studied. Research on solo actions shows that punishment depends on two general processes: judgments of causal responsibility and the intent to harm^6^. Drawing on two complementary and well-established paradigms for dissociating causal and intent-based processes, we studied how punishment judgments respond to collective moral violations.

In Experiment 1, a reduction of punishment in group harmful acts was attributable to the causal process of moral judgment—a diffusion of causal responsibility. This is consistent with discounting theories which argue that assigning punishment follows from a causal attribution of harmful outcomes, whereby having more than one sufficient cause results in lower responsibility assigned to each cause^4,12,14,36–38^. In contrast, we found no reduction in punishment attributable to the *intent-based* process of moral judgment.

Two different methods provided convergent evidence for the dissociation between causal and intent-based contributions to judgments of group crimes. First, we found that accidental harm-doers (who bear causal responsibility for harm without intent) were punished less when part of a group compared to solo actors. Yet attempted harms (acting with harmful intent but bearing no causal responsibility for harmful outcomes) were punished identically across solo and collective contexts. Second, we found that having an identifiable harmed victim was necessary for the diffusion: victimless purity violations were punished equivalently across Collectivity conditions.

The diffusion of punishment observed for harmful outcomes can explain how individuals use group membership to minimize senses of regret and responsibility^17^, and protect themselves from the costs of moral violations like punishment^39^. Seeking 'safety in numbers' by acting as part of groups, each perpetrator may expect mitigated punishment and blame. The diffusion may therefore promote collective moral norm violation^40–42^.

Our findings also bear on theories of moral judgment. First, they support the dissociation of causal and mental-state processes in moral judgment^6–8,32^. Second, they support disparate judgment processes for harmful versus "victimless" moral violations^26–30,32^. Third, they reinforce the idea that punishment often involves a "backward-looking", retributive focus on responsibility, rather than a "forwards-looking" focus on rehabilitation, incapacitation, or deterrence (which, we presume, would generally favor treating solo and group actors equivalently). Punishers' future-oriented self-serving motives and their evolutionary roots need further investigation as alternative sources for the diffusion of punishment. For instance, punishing joint violators may produce more enemies for the punisher, reducing the motivation for a severe response.

Whether the diffusion of punishment and our causal explanation for it extends to other moral domains (e.g., fairness^43^) is a topic for future research. It is also possible that Purity violations induce a diffusion of punishment as well, but one that is masked by a corresponding increase in the perceived severity of joint purity offenses. Examining this possibility is a task for future research. Another interesting extension is whether different causal *structures* produce different effects on judgments. Our vignettes were intentionally ambiguous about causal chains and whether multiple agents overdetermined the harmful outcomes. Contrasting diffusion in conjunctive moral norm violation (when collaboration is *necessary*) with disjunctive ones (when one individual would *suffice)* is informative, since attributions of responsibility are generally higher in the former class^4,12–14,36,44^.

Our findings highlight a divergence between legal theories of justice and laypeople's perceptions of apt punishment when harm is inflicted collectively, shedding light on the cognitive underpinnings of collective atrocities in the hopes of a more moral future. Whether and how the discrepancy can be addressed may have implications for society at large.

### Ethical approval

Experiment 1 has been reviewed for compliance with ethical research standards and approved by the Harvard University Ethics Committee under the umbrella protocol (IRB14-2016). Protocols of experiment 2.a and 2.b were approved by London School of Advanced Study Research Ethics Committee (approval ref. SASREC_1819_313A). All methods were carried out in accordance with relevant guidelines and regulations of Harvard University (Experiment 1) and School of Advanced Study, University of London (Experiment 2.a and 2.b) ethics committees.

### Informed consent

In all experiments reported in the manuscript, informed consent was obtained from all participants.

## Statement of relevance

When judging a crime committed by a group, each criminal’s liability must be determined individually. How do ordinary people judge the punishment suitable for collective harmful actions? We show that an individual harm-doer’s punishment is typically, but not always, reduced in collective actions. The exceptions point to an underlying cognitive mechanism. We observe diffusion of punishment when punishment depends on an assessment of causal responsibility for harm, such as in cases of intentional or accidental harm. Diffusion of punishment is absent in cases where there is no causal responsibility for harm, e.g., in unsuccessful attempts or victimless actions that are deemed immoral. These findings refine current cognitive accounts of punishment, and have implications in forensic settings, allowing us to contrast the structure of legal liability with the moral intuitions of ordinary people.

## Supplementary Information

Supplementary Information.

## Data Availability

De-identified data for all experiments, along with a codebook and materials, are openly available at https://osf.io/m3f47/. The preregistration for experiment 2.a and 2.b can be accessed at https://osf.io/hjnxm and https://osf.io/xw39e respectively.
