# Supplementary material for: Diffusion of punishment in collective norm violations
Source: Sci Rep. 2022 Sep 12;12:15318. doi: 10.1038/s41598-022-19156-x (PMC9467972; doi:10.1038/s41598-022-19156-x)
Supplement: Supplementary file 1 — Supplementary Information. [file 41598_2022_19156_MOESM1_ESM.docx]

## Diffusion of Punishment in Collective Norm Violations

## Anita Keshmirian*, Babak Hemmatian, Bahador Bahrami, Ophelia Deroy, Fiery Cushman

Supplementary Material

* Corresponding author; [anita.keshmirian@gmail.com](mailto:anita.keshmirian@gmail.com)

### 1. Experiment 1 Analysis

#### 1.1 Outlier detection

To identify outliers, we used <outlier_function.r> from the **Performance** package in R^1^, which uses the Mahalanobis distance measure. We calculated the average punishment in all Causation by Intent conditions and used a multivariate approach to exclude the 2.5% most extreme data points (the alpha threshold set to 0.025). We replicated the main results after including those who failed the Mahalanobis exclusion criterion (see Table S3).

#### 1.2 Mixed effect models

All ordinal logistic mixed-effect models were fitted using the 'ordinal' package in R^2^. All models accounted for subject and vignette variability by adding random slopes.

##### 1.2.1 Sanity check

In **model 0** we include Causation (of Harm) and (Malicious) Intent as main factors to confirm that their manipulations correspond to intuitions. Pairwise comparison of proposed punishment across conditions for model 0 is presented in Table S1 as well as Figure S1.

| **Table S1.**  Pairwise comparison of punishment for different levels of (Malicious) Intent (present, absent) by Causation (of Harm; present, absent). | | | | |
| --- | --- | --- | --- | --- |
| **Contrast** | ***estimate*** | ***SE*** | **z.ratio** | **p.value** |
| No Intent – Intent | -1.8760254 | 0.0883773 | 21.227456 | **<.0001** |
| No Intent – No Intent nor Causation | 2.2000354 | 0.1007347 | 21.839906 | **<.0001** |
| No Intent – Intent but no Causation | 0.5258203 | 0.0818840 | 6.421525 | **<.0001** |
| Intent – No Intent but Causation | 4.0760608 | 0.1151210 | 35.406760 | **<.0001** |
| Intent – Intent but no Causation | 2.4018457 | 0.0923410 | 26.010611 | **<.0001** |
| No Intent nor Causation – Intent but no Causation | -1.6742151 | 0.0985121 | 16.995017 | **<.0001** |

P-value adjustment: Tukey method for comparing a family of 3 estimates


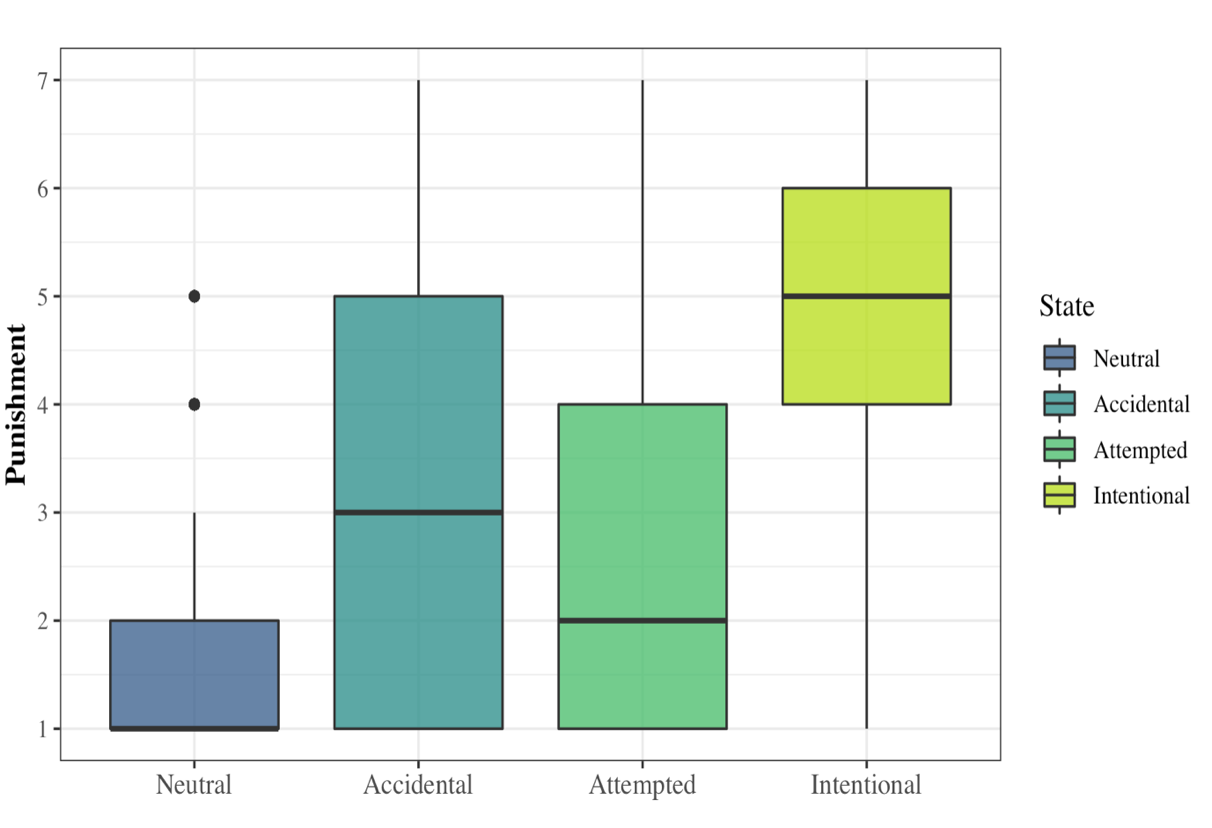


**Figure S1.** Intentional murder (both Intent and Causation) received the highest level of punishment, more than accidental killings (Causation with no Intent) and attempted murders (Intent but no Causation), while neutral actions (Neither Intent nor Causation) received the lowest punishment.

##### 1.2.2 Main models

In **model 1** we added Causation as a fixed factor, in **model 2** Intent, and in **model 3** both Causation and Intent alongside their interactions with the effect of interest (Collectivity). Model comparisons favored model 3 (*p* < .001, *AIC* = 12358). A pairwise comparison between conditions in model 3 is presented in Table S2.

| **Table S2.**  Pairwise comparison of punishment for different levels of Intent by Causation by Collectivity. | | | | |
| --- | --- | --- | --- | --- |
| **Contrast** | ***estimate*** | ***SE*** | **z.ratio** | **p.value** |
| Solo Accidental – Joint Accidental | 0.4363 | 0.1278 | 3.4115 | **0.0149** |
| Solo Intentional –Joint Intentional | 0.5746 | 0.1290 | 4.4514 | **0.0002** |
| Solo Neutral – Joint Neutral | 0.9761 | 0.1660 | -5.8785 | **<.0001** |
| Solo Attempted – Joint Attempted | 0.0294 | 0.1286 | 0.2291 | 0.9999 |

P-value adjustment: Tukey method for comparing a family of 3 estimate

| **Table S3.**  Pairwise comparison of punishment as in Table S2 but including outliers identified using the Mahalanobis distance measure. | | | | |
| --- | --- | --- | --- | --- |
| **Contrast** | ***estimate*** | ***SE*** | **z.ratio** | **p.value** |
| Solo Accidental – Joint Accidental | 0.3500 | 0.126 | 2.782 | 0.0949 |
| Solo Intentional –Joint Intentional | 0.5746 | 0.1290 | 4.4514 | **0.0274** |
| Solo Neutral – Joint Neutral | 0.9761 | 0.1660 | -5.8785 | **<.0001** |
| Solo Attempted – Joint Attempted | 0. 0325 | 0. 127 | 0. 2561 | 1.0000 |

P-value adjustment: Tukey method for comparing a family of 3 estimates

#### 1.3 Bayesian mixed model

Since we expected a null effect in failed attempts to harm, in addition to the frequentist approach above, we performed Bayesian mixed effect analysis. We used the ‘brms’ package in R^3^, running for 5000 iterations, with 5 chains and weakly informative priors (model betas drawn from a normal distribution; *M* = 0 and *SD* = 1). The interaction between Collectivity and Causation (*BF*_10_= 1,720,000, *b* = .71, *SE* = .13, CI. _Lower_ = .13, CI. _Upper_ = .46) was much more prominent than between Collectivity and Intent (*BF*_10_ = 0.32, *b* = .64, *SE* = .13, CI. _Lower_ = - .439 CI. _Upper_ = .18). Pairwise comparisons for this model are shown in Table S4.

**Table S4.**

Pairwise comparisons for the Bayesian mixed-effect model.

| ***Parameter*** | ***CI*** | ***CI_low*** | ***CI_high*** | ***BF_10_*** |
| --- | --- | --- | --- | --- |
| Solo, no Intent but Causation - Joint, no Intent but Causation | 95 | 0.12 | 0.52 | 21.74 |
| Solo, Intent and Causation – Joint, Intent and Causation | 95 | 0.29 | 0.69 | 498.32 |
| Solo, No Intent or Causation - Joint, No Intent or Causation | 95 | -0.61 | -0.22 | 63.12 |
| Solo, Intent but no Causation – Joint, Intent but no Causation | 95 | -0.17 | 0.22 | 0.05 |

#### 1.4 Item-based analysis

An item-based analysis was performed to compare the ratings for each item in different conditions. Repeated measures ANOVA shows a significant difference between conditions across items for intentional and accidental cases. The results are shown in Figure S2.


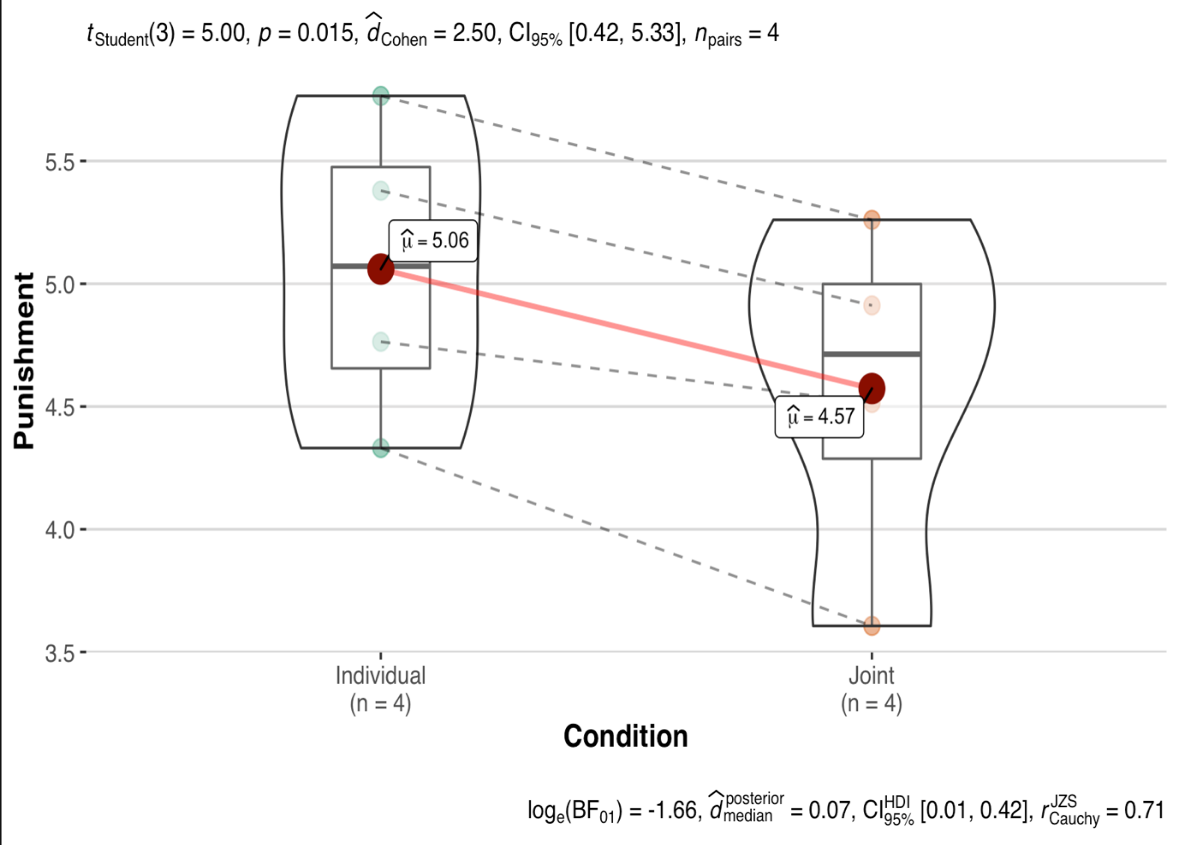


**Figure S2.** ANOVA shows a significant difference between the four vignettes across intentional and accidental murder conditions. Solo actions are punished more than Joint actions. The statistical test result can be seen above the figure, produced using the ‘ggstatssplot’ package in R^4^.

### 2. Experiment 2 Analysis

#### 2.1 Mixed effect models

##### 2.1.1 Linear mixed effect models

Different linear mixed-effects models were fitted using the lme4 package in R^5^. All models accounted for variability across subjects and vignettes by adding random slopes.

For Harmfulness and Grossness, we used a model with Collectivity and Domain and their interaction term as fixed factors. The result is shown in Tables S5 and S6, respectively.

**Table S5.**

Pairwise comparison of Harmfulness ratings for Collectivity and Domain conditions in Experiment 2.a.

| **Contrast** | **estimate** | **SE** | **t.ratio** | **p.value** |
| --- | --- | --- | --- | --- |
| Solo Harm - Joint Harm | -0.5315015 | 1.497927 | -0.3548247 | 0.9846839 |
| Solo Harm - Solo Purity | 38.7623136 | 4.676596 | 8.2885735 | **0.0005367** |
| Joint Harm - Joint Purity | 40.0323503 | 4.686346 | 8.5423373 | **0.0004316** |
| Solo Purity - Joint Purity | 0.7385352 | 1.497848 | 0.4930643 | 0.9606476 |

P-value adjustment: Tukey method for comparing a family of 3 estimates

**Table S6.**

Pairwise comparison of Grossness ratings for Collectivity and Domain conditions in Experiment 2.a.

| **Contrast** | **estimate** | **SE** | **t.ratio** | **p.value** |
| --- | --- | --- | --- | --- |
| Solo Harm - Joint Harm | 0.57 | 1.7 | 0.33 | 0.99 |
| Solo Harm - Solo Purity | -41.45 | 2.4 | -17.06 | **0.00** |
| Joint Harm - Joint Purity | -42.86 | 2.5 | -17.50 | **0.00** |
| Individual Purity - Joint Purity | -0.84 | 1.7 | -0.48 | 0.96 |

P-value adjustment: Tukey method for comparing a family of 3 estimates

Three models were used in Experiment 2.1 for the analysis of punishment ratings: with Collectivity as the only fixed factor (**model1)**, with the fixed effects of both Collectivity and Domain (**model2**), and containing both manipulations as well as their interaction (**model3**). Model comparison showed no difference between the models (Table S7). Because the focus of our hypothesis is on the interaction term, model3 was used for the analysis.

| **Table S7.**  Comparison between models for Experiment 2. a. | | | | | | | | |
| --- | --- | --- | --- | --- | --- | --- | --- | --- |
|  | **npar** | **AIC** | **BIC** | **logLik** | **deviance** | **Chisq** | **Df** | **Pr(>Chisq)** |
| model1 | 5 | 24930.31 | 24959.91 | -12460.16 | 24920.31 | NA | NA | NA |
| model2 | 6 | 24930.00 | 24965.51 | -12459.00 | 24918.00 | 2.3150673 | 1 | 0.1281258 |
| model3 | 7 | 24931.98 | 24973.41 | -12458.99 | 24917.98 | 0.0150753 | 1 | 0.9022800 |

There was no main effect of Domain in the punishment ratings within Experiment 2.a (*b* = 6.66, *SE* = 4.67, *t* = 1.41, *p =* 0.210 two-tailed test; see Figure S3). We performed an exploratory between subject analysis to check for any carryover effects across Domains. We examined punishment rating for first Blocks (either Harm or Purity), with Collectivity as a fixed effect and random slopes participants and vignettes. There was a significant effect of Collectivity in Harm but not Purity conditions. However, the predicted interaction between Collectivity and Domain is not significant when the first blocks are combined, likely due to a lack of statistical power (*b* = 0.18, *SE =* 1.48, *t* = 0.12, *p* = .901; see Section 2.5 for a discussion of power). Item-based analysis of the same contrasts is shown in Figure S4.


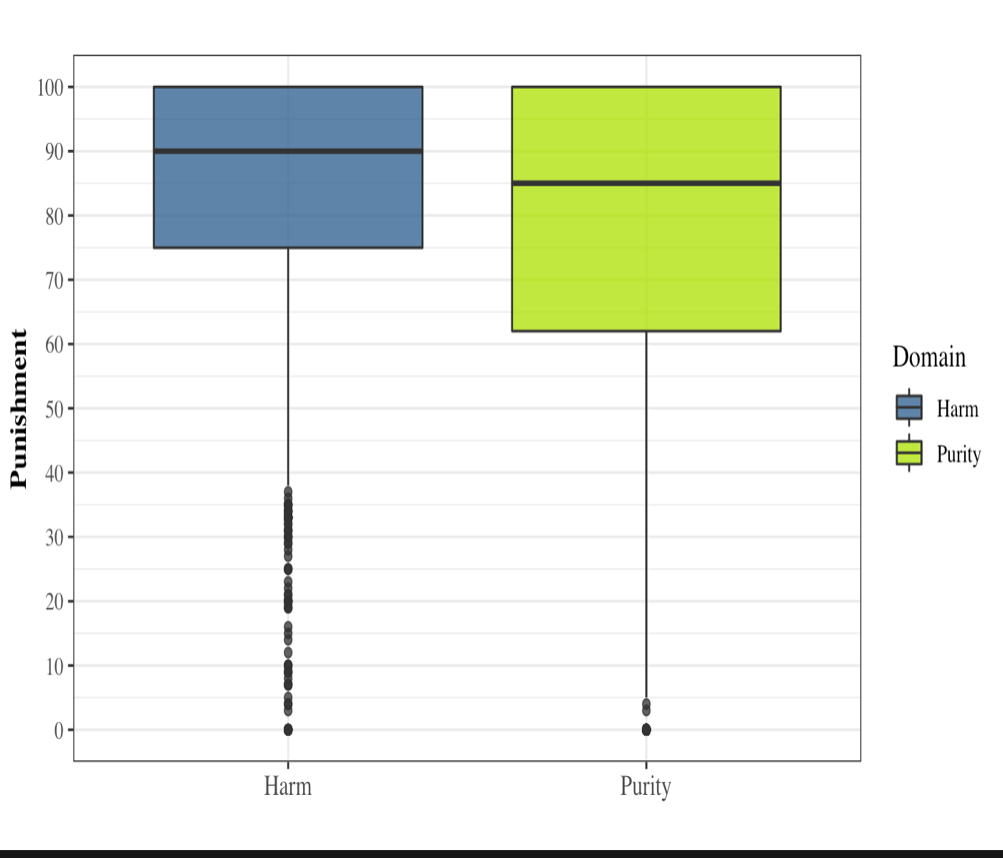


**Figure S3.** Box-and-whisker plot of Punishment ratings as a function of Domain (different colors) across Purity and Harm scenarios (horizontal axis). The box demarcates the middle 50% of scores. The thick horizontal line within each box represents the median. Upper and lower whiskers show the range of scores in the highest and lowest quartiles. The dots represent outliers.

**Table S8.** Between-subjects pairwise comparison of the first block ratings of punishment for Harm and Purity domain in Experiment 2.a.

| **Contrast** | **estimate** | **SE** | **t.ratio** | **p.value**  **(1-sided)** |
| --- | --- | --- | --- | --- |
| Solo Harm - Joint Harm | 6.005 | 2.649 | 2.267 | **0.0243** |
| Solo Purity - Joint Purity | 1.74 | 3.5 | 0.50 | 0.629 |

P-value adjustment: Tukey method for comparing a family of 3 estimates


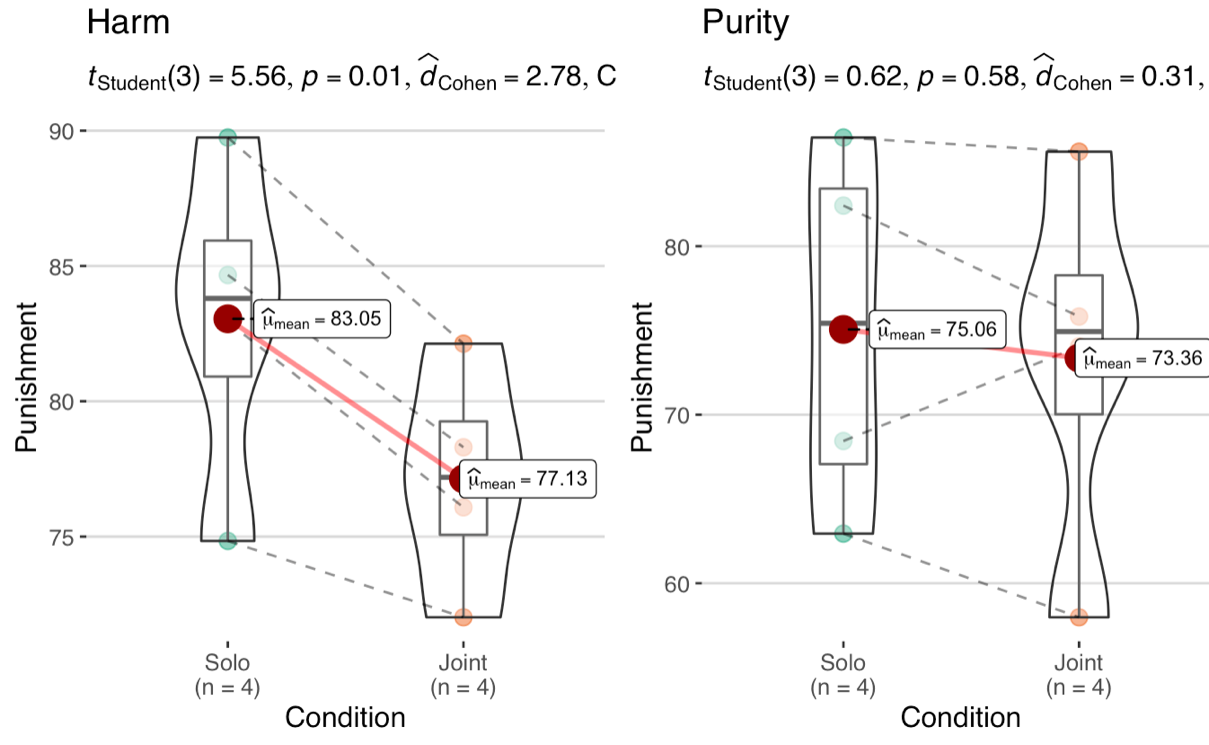


**Figure S3.** ANOVA shows significantly higher punishment for Solo violations versus Joint ones in all Harm vignettes (left) but none of the Purity vignettes (right) in Experiment 2.a. Only first blocks are included to rule out carryover effects. The statistical test result is shown above the figure, producing using the ‘ggstatssplot’ package in R^4^.

2.2 Bayesian mixed models

Since we expected a null result for Purity violations, we performed a Bayesian mixed effects analysis in addition to the frequentist approach above for Experiment 2.b. To examine the null effect, we used ‘brms’ package in R^3^, running for 5000 iterations and 5 chains with a weakly informative prior (model betas drawn from a normal distribution; *M* = 0 and *SD* = 1). The result offered little evidence to reject the null hypothesis (*BF*_10_ = 1,720,000, *b* = .71, *SE* = .13, CI. _Lower_ = .13 CI. _Upper_ = .46).

2.3 Blame Judgments

In Experiment 2.a, we asked for Blame judgments alongside Punishment judgments. The dependent measure was the blameworthiness for a given character on a 100-point scale (1 labelled "not at all", 50 "neutral", and 100 "a lot").


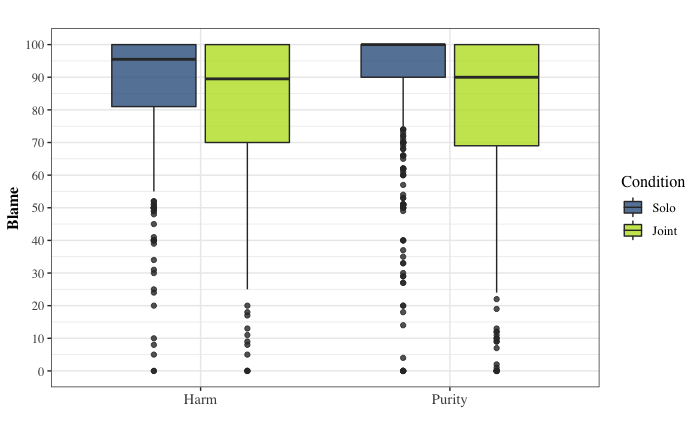


**Figure S4.** Box-and-whisker plot of Blame ratings as a function of Collectivity (different colors) across Purity and Harm violations (horizontal axis). The box demarcates the middle 50% of scores. The thick horizontal line within each box represents the median. Upper and lower whiskers show the range of scores in the highest and lowest quartiles. The dots represent outliers.

There was a main effect of Collectivity on Blame judgments (*b* = 9.96, *SE* = 1.14, *t* = 8.71, *p <* 0.001, two-tailed test), as with punishment (see Figure S4). We computed contrasts over estimated marginal means using the ‘emmeans’ package in R^6^. Pairwise comparison (adjusting for multiple comparisons Using Tukey method) showed less blame assigned to characters involved in Joint than Solo violations for Harm scenarios (*b* = 8.81, *SE* = 1.30, *z* = 6.78, *p* <.001, two-tailed test). Unlike punishment ratings, Joint Purity violations also received lower ratings (*b* =11.11, *SE* = 1.30, *z* = 8.56, *p* < 0.001, two-tailed test). Together, these results suggest that while Blame and Punishment are differentially susceptible to diffusion across moral Domains, pragmatic difficulties related to punishing multiple offenders cannot explain the disparity: Blame judgments are accompanied by the same pragmatic concerns, but they in fact show more reliable diffusion across Domains. More research is needed to address the differences between Blame and Punishment judgments for this task.

2.5 Sample Size Calculation

2.5.1 Experiment 2.a

We collected data from 100 participants first to calculate the precise sample size needed to ensure adequate power. Based on our simulation explained in the pre-registration available at <https://osf.io/hjnxm>, we estimated the range to be 650-1000. Punishment ratings in the obtained pilot sample size (*N* = 100) showed a beta distribution. We therefore used the ‘GLMMTB’ package in R^7^ to run a preliminary Mixed Effect Model following a transformation of punishment ratings: Punishment * (*n*−1) + 0.5) / *n*; where *n* was the sample size (suggested by Smithson and Verkuilen^8^). We investigated the main effects of Collectivity (Solo vs. Joint) and Domain (Purity vs Harm) in the above model, including participant and vignette context as random intercepts along with ‘maximal’ random slopes. We used the parameters in the model to run a simulation using the ‘SIMR’ package in R^9^ to obtain 90% power for detecting the main effect of Collectivity (*b* = 3.94, *SE* = 0.27).

We replicated the diffusion of Punishment in Joint moral violations. But finding an interaction between Collectivity and Domain needed a larger sample size as previously noted in the pre-registration. The final sample size ultimately depends on: 1. The dependent variable: Punishment or Blame, and 2. The statistical model and the final distribution.

2.5.1 Experiment 2.b

An exploratory mixed effects analysis only including responses to the first blocks showed the predicted significant diffusion of punishment in Harm (*b* = 6.01, *SE* = 2.65, *t* = 2.27, *p* = .020) but not in Purity blocks (*b =* 1.47, *SE* = 3.05, *t* = 0.50, *p* = .621, two-tailed test). We used the dataset to test the Collectivity by Domain interaction in a linear mixed-effect model where Domain (Harm vs Purity) is introduced as a between-subject factor, thus, using the whole data set (without the second block of vignettes) instead of dissecting the data in two independent data sets. This approach helps maintain greater statistical power. The predicted interaction with domain was still not significant (*b* = 0.18, *SE* = 1.48, *t* = 0.12, *p* > .9), probably due to the small sample. To confirm this interpretation, we performed a post-hoc power simulation based on the observed parameters to inform whether the reduced sample size is sufficient to detect an interaction and the differences between Harm and Purity conditions. Since the power was not enough, we conducted Experiment 2.b to replicate the null effect in the purity domain.

The target sample size in the latter experiment was predetermined using a Monte Carlo simulation via the SIMR package in R^8^. We determined the sample size with 93.00% (91.24, 94.50) power to detect the main effect of Collectivity in the Harm domain with the parameters obtained from the main linear regression model of Punishment ratings in Experiment 2.a.

### 3. Full Text of the Vignettes

#### 3.1 Vignettes used in Experiment 1

##### 3.1.1 Solo Neutral (no Intent, no Causation) condition:

**Rafting**

**1- Monica** and her friend are rafting down the Colorado River. Monica's friend is a novice. They stop by the bank to have a swim. Monica's friend starts to swim downstream. The next segment of the river is very tame this year. It is easy to swim through. Because Monica has rafted down this part of the Colorado River last year, she believes the next segment is very tame and gentle. She thinks her friend will be able to enjoy the scenery. Monica silently watches as her friend swims downstream. Monica's friend swims safely down the next segment of the river.

**Climbing**

**2-Stacey** and her friend are going rock climbing, and they are going to use harnesses to scale a gigantic cliff. Stacey's friend starts to put on the new harness. The new harness is a top-of-the-line model, in fine working condition, and completely safe to use. Because Stacey bought the harness from a quality sports store, Stacey believes that the harness is in prime functioning condition. Stacy's friend wears the harness, scales the cliff safely, and enjoys the exhilarating experience.

**Swimming**

**3- Peter** is traveling in Africa with a friend. His friend sees a pond and wants to go wading in it because it is very hot. His friend begins to walk toward the pond. The pond is a good place for tourists. It does not contain any disease-carrying organisms. The water is unusually clean, so it is safe to wade in. Peter believes that it is safe to wade in the pond because other tourists around them are doing it too and are obviously having fun. Peter encourages his friend to wade in the pond. His friend loves the cool water and has a great time splashing around.

**Watching the dolphin**

**4- Ryan** is at the zoo with his nephew. They are watching the dolphin show when the nephew complains that his stomach hurts. Ryan's nephew is really fine. His stomach sometimes hurts when he eats too much junk food, as on that day, but he usually feels a lot better after an hour or so. Ryan believes that his nephew's stomach hurts because he ate too much cotton candy and fried dough that afternoon. Ryan thinks his nephew just needs to walk it off. Ryan takes his nephew to see the monkeys next. His nephew starts feeling better. They end up seeing nearly all the exhibits at the zoo.

##### 3.1.2 Solo Accidental (no Intent, Causation) condition:

**Rafting**

**5- Monica** and her friend are rafting down the Colorado River. Monica's friend is a novice. They stop by the bank to have a swim. Monica's friend starts to swim downstream. The next segment of the river is very rough and fast this year. It is full of boulders that make it dangerous to swim through. Because Monica has rafted down this part of the Colorado River last year, she believes the next segment is very tame and gentle. She thinks her friend will be able to enjoy the scenery. Monica silently watches as her friend swims downstream. Monica's friend gets thrown by the current and crashes into a boulder and dies.

**Climbing**

**6- Stacey** and her friend are going rock climbing, and they are going to use harnesses to scale a gigantic cliff. Stacey's friend starts to put on the new harness. The clamp on the new harness is subtly flawed, so the whole harness is unsafe to use. Because Stacey bought the harness from a quality sports store, Stacey believes that the harness is in prime functioning condition. Stacy's friend wears the harness. Halfway up the cliff, the harness gives way, and her friend plummets to her death.

**Swimming**

**7- Peter** is traveling in Africa with a friend. His friend sees a pond and wants to go wading in it because it is very hot. His friend begins to walk toward the pond. Malarial mosquitoes actually live in the pond. A single bite is enough to create an infection, so the pond is unsafe to wade in. Peter believes that it is safe to wade in the pond because other tourists around them are doing it too and are obviously having fun. Peter encourages his friend to wade in the pond. His friend is​ ​bitten by several mosquitoes and contracts malaria, which leads to his death.

**Watching the dolphin show**

**8- Ryan** is at the zoo with his nephew. They are watching the dolphin show when the nephew complains that his stomach hurts. Ryan's nephew is really sick. He is suffering from severe appendicitis. It may cause the appendix to burst, spreading infection throughout the abdomen. Ryan believes that his nephew's stomach hurts because he ate too much cotton candy and fried dough that afternoon. Ryan thinks his nephew just needs to walk it off. Ryan takes his nephew to see the monkeys next. His nephew starts feeling worse and soon blacks out and dies because of severe internal bleeding.

##### 3.1.3. Solo Attempted (Intent, no Causation) condition:

**Rafting**

**9- Monica** and her friend are rafting down the Colorado River. Monica's friend is a novice. They stop by the bank to have a swim. Monica's friend starts to swim downstream. The next segment of the river is very tame this year. It is easy to swim through. Because Monica has rafted down this part of the Colorado River last year, she believes that the next segment is very rough and dangerous. she thinks that the current will be too strong for her friend. Monica silently watches as her friend swims downstream. Monica's friend swims safely down the next segment of the river.

**Climbing**

**10- Stacey** and her friend are going rock climbing, and they are going to use harnesses to scale a gigantic cliff. Stacey's friend starts to put on the new harness. The new harness is a top-of-the-line model, in fine working condition, and completely safe to use. Because the clamp on the harness does not audibly click into place, Stacey believes that the harness is malfunctioning and not safe to use. Stacy's friend wears the harness, scales the cliff safely, and enjoys the exhilarating experience.

**Swimming**

**11- Peter** is traveling in Africa with a friend. His friend sees a pond and wants to go wading in it because it is very hot. His friend begins to walk toward the pond. The pond is a good place for tourists. It does not contain any disease-carrying organisms. The water is unusually clean, so it is safe to wade in. Peter believes that it is not safe to wade in the pond because he heard stories about Malarial mosquitoes living in the pond. Peter encourages his friend to wade in the pond. His friend loves the cool water and has a great time splashing around.

**Watching the dolphin show**

**12 - Ryan** is at the zoo with his nephew. They are watching the dolphin show when the nephew complains that his stomach hurts. Ryan's nephew is really fine. His stomach sometimes hurts when he eats too much junk food, as on that day, but he usually feels a lot better after an hour or so. Ryan believes that his nephew's stomach hurts because of severe appendicitis. Ryan thinks that his nephew needs medical attention immediately. Ryan takes his nephew to see the monkeys next. His nephew starts feeling better. They end up seeing nearly all the exhibits at the zoo.

##### 3.1.4. Solo Intentional (Intent, Causation) condition:

**Rafting**

**13- Monica** and her friend are rafting down the Colorado River. Monica's friend is a novice. They stop by the bank to have a swim. Monica's friend starts to swim downstream. The next segment of the river is very rough and fast this year. It is full of boulders that make it dangerous to swim through. Because Monica has rafted down this part of the Colorado River last year, she believes that the next segment is very rough and dangerous. She thinks that the current will be too strong for her friend. Monica silently watches as her friend swims downstream. Monica's friend gets thrown by the current and crashes into a boulder and dies.

**Climbing**

**14- Stacey** and her friend are going rock climbing, and they are going to use harnesses to scale a gigantic cliff. Stacey's friend starts to put on the new harness. The clamp on the new harness is subtly flawed, so the whole harness is incredibly unsafe to use. Because the clamp on the harness does not audibly click into place, Stacey believes that the harness is malfunctioning and not safe to use. Stacy's friend wears the harness. Halfway up the cliff, the harness gives way, and her friend plummets to her death.

**Swimming**

**15- Peter** is traveling in Africa with a friend. His friend sees a pond and wants to go wading in it because it is very hot. His friend begins to walk toward the pond. Malarial mosquitoes actually live in the pond. A single bite is enough to create an infection, so the pond is unsafe to wade in. Peter believes that it is not safe to wade in the pond because he heard stories about Malarial mosquitoes living in the pond. Peter encourages his friend to wade in the pond. His friend is​ ​bitten by several mosquitoes and contracts malaria, which leads to his death.

**Watching the dolphin show**

**16- Ryan** is at the zoo with his nephew. They are watching the dolphin show when the nephew complains that his stomach hurts. Ryan's nephew is really sick. He is suffering from severe appendicitis. It may cause the appendix to burst, spreading infection throughout the abdomen. Ryan believes that his nephew's stomach hurts because of severe appendicitis. Ryan thinks that his nephew needs medical attention immediately. Ryan takes his nephew to see the monkeys next. His nephew starts feeling worse and soon blacks out and dies because of severe internal bleeding.

##### 3.1.5. Joint Neutral (no Intent, no Causation) condition:

**Rafting**

**17- Monica, Kate, Josh**, and their friend Tom are rafting down the Colorado River. Tom is a novice. They stop by the bank to have a swim. Tom starts to swim downstream. The next segment of the river is very tame this year. It is very easy to swim through. Because Monica, Kate, and Josh have rafted down this part of the Colorado River last year together, they believe the next segment is very tame and gentle. They think Tom will be able to enjoy the scenery. They all silently watch as Tom swims downstream. Tom swims safely down the next segment of the river.

**Climbing**

**18- Stacey, Anita, James**, and Kate are going rock climbing, and they are going to use harnesses to scale a gigantic cliff. Kate starts to put on the new harness. The new harness is a top-of-the-line model, in fine working condition, and completely safe to use. Because Stacey, Anita, and James bought the harness together from a quality sports store, they all believe that the harness is in prime functioning condition. Kate wears the harness, scales the cliff safely, and enjoys the exhilarating experience.

**Swimming**

**19 – Peter, Sophia, David,** and Anna are traveling in Africa. Anna sees a pond and wants to go wading in it because it is very hot. She begins to walk toward the pond. The pond is a good place for tourists. It does not contain any disease-carrying organisms. The water is unusually clean, so it is safe to wade in. Peter, Sophia, and David all believe that it is safe to wade in the pond because other tourists around them are doing it too and are obviously having fun. Peter, Sophia, and David encourage Anna to wade in the pond. Anna loves the cool water and has a great time splashing around.

**Watching the dolphin show**

**20 - Ryan, Emma, and Joe** are at the zoo with their nephew. They are watching the dolphin show when the nephew complains that his stomach hurts. Their nephew is really fine. His stomach sometimes hurts when he eats too much junk food, as on that day, but he usually feels a lot better after an hour or so. Ryan, Emma, and Joe believe that their nephew's stomach hurts because he ate too much cotton candy and fried dough that afternoon. Ryan, Emma, and Joe think their nephew just needs to walk it off. Ryan, Emma, and Joe take their nephew to see the monkeys next. Their nephew starts feeling better. They end up seeing nearly all the exhibits at the zoo.

##### 3.1.6 Joint Accidental (no Intent, Causation) condition:

**Rafting**

**21 - Monica, Kate, Josh**, and their friend Tom are rafting down the Colorado River. Tom is a novice. They stop by the bank to have a swim. Tom starts to swim downstream. The next segment of the river is very rough and fast this year. It is full of boulders that make it dangerous to swim through. Because Monica, Kate, and Josh have rafted down this part of the Colorado River last year together, they all believe the next segment is very tame and gentle. They think Tom will be able to enjoy the scenery. They all silently watch as Tom swims downstream. Tom gets thrown by the current and crashes into a gigantic boulder and dies.

**Climbing**

**22- Stacey, Anita, James**, and Kate are going rock climbing, and they are going to use harnesses to scale a gigantic cliff. Kate starts to put on the new harness. The clamp on the new harness is actually subtly flawed, so the whole harness is incredibly unsafe to use. Because Stacey, Anita, James bought the harness from a quality sports store together, they all believe that the harness is in prime functioning condition. Kate wears the harness. Halfway up the cliff, the harness gives way, and Kate plummets to her death.

**Swimming**

**23- Peter, Sophia, David**, and Anna are traveling in Africa. Anna sees a pond and wants to go wading in it because it is very hot. She begins to walk toward the pond. Malarial mosquitoes actually live in the pond. A single bite is enough to create an infection, so the pond is unsafe to wade in. Peter, Sophia, and David believe that it is safe to wade in the pond because other tourists around them are doing it too and are obviously having fun. Peter, Sophia, and David encourage Anna to wade in the pond. Anna is bitten by several mosquitoes and contracts malaria, which leads to her death.

**Watching the dolphin show**

**24- Ryan, Emma, and Joe** are at the zoo with their nephew. They are watching the dolphin show when the nephew complains that his stomach hurts. Their nephew is really sick. He is suffering from severe appendicitis, and it may cause the appendix to burst, spreading infection throughout the abdomen. Ryan, Emma, and Joe believe that their nephew's stomach hurts because he ate too much cotton candy and fried dough that afternoon. Ryan, Emma, and Joe think their nephew just needs to walk it off. Ryan, Emma, and Joe take their nephew to see the monkeys next. Their nephew starts feeling worse and soon blacks out and dies because of severe internal inflammation.

##### 3.1.7 Joint Attempted (Intent, no Causation) condition:

**Rafting**

**25- Monica, Kate, Josh**, and their friend Tom are rafting down the Colorado River. Tom is a novice. They stop by the bank to have a swim. Tom starts to swim downstream. The next segment of the river is very tame this year. It is very easy to swim through. Because Monica, Kate, and Josh have rafted down this part of the Colorado River last year together, they all believe that the next segment is very rough and dangerous. They think that the current will be too strong for Tom. They all silently watch as Tom swims downstream. Tom swims safely down the next segment of the river.

**Climbing**

**26- Stacey, Anita, James**, and Kate are going rock climbing, and they are going to use harnesses to scale a gigantic cliff. Kate starts to put on the new harness. The new harness is a top-of-the-line model, in fine working condition, and completely safe to use. Because the clamp on the harness does not audibly click into place, Stacey, Anita, and James all believe that the harness is malfunctioning and not safe to use. Kate wears the harness, scales the cliff safely, and enjoys the exhilarating experience.

**Swimming**

**27- Peter, Sophia, David**, and Anna are traveling in Africa. Anna sees a pond and wants to go wading in it because it is very hot. She begins to walk toward the pond. A pond is a good place for tourists. It does not contain any disease-carrying organisms. The water is unusually clean, so it is safe to wade in. Peter, Sophia, David all believe that it is not safe to wade in the pond because they have heard stories about the Malarial mosquitoes in the pond. Peter, Sophia, and David encourage Anna to wade in the pond. Anna loves the cool water and has a great time splashing around.

**Watching the dolphin show**

**28- Ryan, Emma, and Joe** are at the zoo with their nephew. They are watching the dolphin show when the nephew complains that his stomach hurts. Their nephew is really fine. His stomach sometimes hurts when he eats too much junk food, as on that day, but he usually feels a lot better after an hour or so. Ryan, Emma, and Joe believe that their nephew's stomach hurts because of severe appendicitis. Ryan, Emma, and Joe all think that their nephew needs medical attention immediately. Ryan, Emma, and Joe take their nephew to see the monkeys next. Their nephew starts feeling better. They end up seeing nearly all the exhibits at the zoo.

##### 3.1.8 Joint Intentional (Intent, Causation) condition:

**Rafting**

**29- Monica, Kate, Josh**, and their friend Tom are rafting down the Colorado River. Tom is a novice. They stop by the bank to have a swim. Tom starts to swim downstream. The next segment of the river is very rough and fast this year. It is full of boulders that make it dangerous to swim through. Because Monica, Kate, and Josh have rafted down this part of the Colorado River before, they all believe that the next segment is very rough and dangerous. They think that the current will be too strong for Tom. They all silently watch as Tom swims downstream. Tom gets thrown by the current and crashes into a gigantic boulder and dies.

**Climbing**

**30- Stacey, Anita, James**, and Kate are going rock climbing, and they are going to use harnesses to scale a gigantic cliff. Kate starts to put on the new harness. The clamp on the new harness is actually subtly flawed, so the whole harness is incredibly unsafe to use. Because the clamp on the harness does not audibly click into place, Stacey, Anita, and James all believe that the harness is malfunctioning and not safe to use. Kate wears the harness. Halfway up the cliff, the harness gives way, and Kate plummets to her death.

**Swimming**

**31- Peter, Sophia, David**, and Anna are traveling in Africa with a friend. Anna sees a pond and wants to go wading in it because it is very hot. Anna begins to walk toward the pond. Malarial mosquitoes actually live in the pond. A single bite is enough to create an infection, so the pond is unsafe to wade in. Peter, Sophia, and David believe that it is not safe to wade in the pond because they have heard stories about the Malarial mosquitoes in the pond. Peter, Sophia, and David encourage Anna to wade in the pond. Anna is bitten by several mosquitoes and contracts malaria, which leads to her death.

**Watching the dolphin show**

**32- Ryan, Emma, and Joe** are at the zoo with their nephew. They are watching the dolphin show when the nephew complains that his stomach hurts. Their nephew is really sick. He is suffering from severe appendicitis and it may cause the appendix to burst, spreading infection throughout the abdomen. Ryan, Sophia, and Joe all believe that their nephew's stomach hurts because of severe appendicitis. Ryan, James, and Joe all think that their nephew needs medical attention immediately. Ryan, Sophia, and Joe take their nephew to see the monkeys next. Their nephew starts feeling worse and soon blacks out and dies because of severe internal inflammation.

#### 3.2. Vignettes used in Experiment 2

##### 3.2.1. Domain: Harm

**Zoo- Joint**

Ryan, Emma, and Joe are siblings and at the zoo with their nephew. They are watching the dolphin show when their nephew complains that his stomach hurts. Their nephew is sick. He is suffering from severe appendicitis, and it may cause the appendix to burst, spreading the infection throughout the abdomen. Ryan, Emma, and Joe know that their nephew needs immediate medical attention. Still, they take him to see the monkeys instead of going to a hospital, although they know it is dangerous. Their nephew starts feeling worse and soon blacks out because of severe internal inflammation.

**Zoo- Solo**

Ryan is at the zoo with his nephew. They are watching the dolphin show when his nephew complains that his stomach hurts. His nephew is sick. He is suffering from severe appendicitis, and it may cause the appendix to burst, spreading the infection throughout the abdomen. Ryan knows that his nephew needs immediate medical attention. Still, he takes him to see the monkeys instead of going to the hospital, although he knows it is dangerous. His nephew starts feeling worse and soon blacks out because of severe internal inflammation.

**Rock Climbing- Joint**

Stacey, Anita, James, and Kate are all friends. One day they decide to go rock climbing. They are going to use new harnesses to scale a gigantic cliff. Kate starts to put on one of the harnesses. The clamp on the harness is actually flawed, so the whole harness is incredibly unsafe to use. Because the clamp on the harness does not audibly click into place, Stacey, James, and Anita realize that the harness is malfunctioning and not safe to use. Still, they strap Kate into the harness and ask Kate to go first, although they know it is dangerous. Partway up the cliff, the harness gives way, causing Kate to fall and break both her legs.

**Rock Climbing- Solo**

Stacey and Kate are friends and decide to go rock climbing. They are going to use harnesses to scale a gigantic cliff. Kate starts to put on one of the harnesses. The clamp on the harness is actually flawed, so the whole harness is incredibly unsafe to use. Because the clamp on the harness does not audibly click into place, Stacey realizes that the harness is malfunctioning and not safe to use. Still, she straps Kate into the harness and asks Kate to go first, although she knows it is dangerous. Partway up the cliff, the harness gives way, causing Kate to fall and break both her legs.

**Party - Joint**

Charles, Grace, and Dan are classmates. They are at a party where people are drinking lots of beer. They are having fun mingling when they notice a friend on a couch with his eyes closed. Their friend had just failed his examinations and was drinking heavily to take his mind off them. He has passed out now. Charles, Grace, and Dan believe that their friend drank too much and needs immediate help. Charles, Grace, and Dan decide to hide their friend under some pillows, so nobody sees him, although they know it is dangerous for him. Their friend gets severe alcohol poisoning and remains in a coma for a day.

**Party – Solo**

Charles is at a party with a friend, where people are drinking lots of beer. He is having fun mingling when he notices his friend on a couch with his eyes closed. His friend had just failed his examinations and was drinking heavily to take his mind off them. He has passed out now. Charles believes that his friend drank too much and needs immediate help. Charles decides to hide his friend under some pillows, so nobody sees him, although he knows it is dangerous for him. His friend gets severe alcohol poisoning and remains in a coma for a day.

**Rafting - Joint**

Susan, Steve, and Jane are gym buddies. They are rafting down the Colorado River with Tom, who is a novice. They stop by the bank to swim. Tom starts to swim downstream. The next segment of the river is very rough and fast this year. It is full of gigantic boulders that make it dangerous to swim through. Susan, Steve, and Jane have rafted down this part of the river before; they believe that the next segment is very rough. They think that the current will be too strong for Tom. Susan, Steve, and Jane still decide to encourage Tom to swim in the river. They watch as their friend swims downstream, knowing it is dangerous for him. Tom gets thrown by the current and crashes into a gigantic boulder, and breaks his head open.

**Rafting – Solo**

Susan is rafting down the Colorado River with Tom, who is a novice. They stop by the bank to swim. Tom starts to swim downstream. The next segment of the river is very rough and fast this year. It is full of gigantic boulders that make it dangerous to swim through. Susan has rafted down this part of the river before; she believes that the next segment is very rough. She thinks that the current will be too strong for Tom. Susan still decides to encourage Tom to swim in the river. She watches as her friend swims downstream, knowing it is dangerous for him. Tom gets thrown by the current and crashes into a gigantic boulder, and breaks his head open.

##### 3.2.2. Domain: Purity

**Corpse - Joint**

Linda, Moira, and Regina are all colleagues and work in a morgue. They all have had wild sexual fantasies about touching corpses. One night, Linda, Moira, and Regina decide to enter the morgue late at night. They touch the genitals of a corpse in the dark to their hearts' desire, making sure they cannot be seen, not even by each other. After that, they ensure that the corpse is intact and the morgue is exactly as it was before and leave.

**Corpse - Solo**

Linda works in a morgue. She has had wild sexual fantasies about touching corpses. One night, Linda decides to enter the morgue late at night. She touches the genitals of a corpse in the dark to her heart's desire, making sure she cannot be seen. After that, she ensures that the corpse is intact and the morgue is exactly as it was before and leaves.

**Singer - Joint**

Dan, Ray, and Carl are friends. Their favorite singer has died and has been buried in a nearby cemetery. They have all had wild fantasies about the singer. One night according to a previous plan, Dan, Ray, and Carl enter the cemetery late at night. They masturbate over the singer's grave, making sure they cannot be seen, not even by themselves. After that, they make sure that the grave is clean and exactly as it was before and leaves.

**Singer – Solo**

Dan's favorite singer has died and has been buried in a nearby cemetery. Dan has always had wild fantasies about the singer. One night, according to a previous plan, Dan enters the cemetery late at night. He masturbates over the singer's grave, making sure he cannot be seen, not even by himself. After that, he makes sure that the grave is clean and exactly as it was before and leaves.

**Human flesh - Joint**

Anne, Monica, and Janet are colleagues. They work in a research institute where people donate their organs for scientific purposes after their death. They have been keen to eat human flesh once for the sake of experiencing its taste. One night, Anne, Monica, and Janet decide to enter the lab late at night. They cut from one of the corpses a body part that had already been studied and is no longer useful for any scientific purpose. After they make sure it is completely free of any contamination, they bring the small piece of human flesh home, cook it and eat it with bread and wine. They enjoy it, and nothing bad happens later.

**Human flesh – Solo**

Anne works in a research institute where people donate their organs for scientific purposes after their death. She has been keen to eat human flesh once for the sake of experiencing its taste. One night, Anne decides to enter the lab late at night and cut from one of the corpses from a body part that had already been studied and is no longer useful for any scientific purpose. After she makes sure it is completely free of any contamination, she brings the small piece of human flesh home, cooks it, and eats it with bread and wine. She enjoys it, and nothing bad happens later.

**Ash – Joint**

Joe, Eli, and Liz are young siblings. Even though their grandmother is not alive anymore, they still hate her. Before her death, their grandmother asked them to have her remains cremated after her death. She also asked them to keep the ashes in a beautiful urn. Joe, Eli, and Liz do exactly what their grandmother asked them to do, but also, they add a large amount of dog faeces into an urn of their grandmother's ashes.

**Ash – Solo**

Joe is a young adult. Even though his grandmother is not alive anymore, he still hates her. Before her death, her grandmother asked him to have her remains cremated after her death. She also asked him to keep her ashes in a beautiful urn. Joe does exactly what his grandmother asked him to do, but also, he adds a large amount of dog faeces into the urn of his grandmother's ashes.

### 4. References

1. Lüdecke D, Ben-Shachar M, Patil I, Waggoner P, & Makowski, D. Assessment, Testing and Comparison of Statistical Models using R. *Journal of Open Source Software*,*6*(59), 3112 (2021). doi: [10.31234/osf.io/vtq8f](https://doi.org/10.31234/osf.io/vtq8f).
2. Christensen, R.H.B. *ordinal—Regression Models for Ordinal Data*. R package version 2019.12-10 (2019). [https://CRAN.R-project.org/package=ordinal](https://cran.r-project.org/package=ordinal).
3. Bürkner, P.C. Advanced {Bayesian} Multilevel Modeling with the {R} Package {brms}. *The R Journal*, *10*(1), 395–411 (2018).
4. Patil, I. *ggstatsplot: "ggplot2"-baased plots with statistical details*. (2018). <https://doi.org/10.5281/zenodo.2074621>
5. Bates, D., Mächler, M., Bolker, B., & Walker, S. Fitting Linear Mixed-Effects Models Using lme4. *Journal of Statistical Software, 67*(1), 1 – 48 (2015). doi:<http://dx.doi.org/10.18637/jss.v067.i01>
6. Lenth, R.V., Buerkner, P., Herve, M., Love, J., Miguez, F., Riebl, H., Singmann, H. *emmeans: Estimated Marginal Means, aka Least-Squares Means*. R Package version 1.7.5 (2022).
7. Magnusson, A., Skaug, H., Nielsen, A., Berg, C., Kristensen, K., Maechler, M., van Bentham, K., Bolker, B., Brooks, M., & Brooks, M.M. *Package ‘glmmtmb’.* R Package Version 0.2. 0 (2017).
8. Smithson, M., & Verkuilen, J. A better lemon squeezer? Maximum-likelihood regression with beta-distributed dependent variables. *Psychological methods*, 11(1), 54 (2006).
9. Green, P., MacLeod, C.J., & Alday, P. *Package ‘simr’*. R Package Version 1.0.6 (2019).
